# The association of global longitudinal and global circumferential strain with ejection fraction in patients with aortic stenosis and increased left ventricular mass: a feature tracking CMR study

**DOI:** 10.1186/1532-429X-17-S1-P226

**Published:** 2015-02-03

**Authors:** Joao L Cavalcante, Antonia Delgado-Montero, Shasank Rijal, Erik B Schelbert, John Gorcsan III

**Affiliations:** Department of Medicine, University of Pittsburgh School of Medicine, Kragujevac, PA USA; Cardiovascular Magnetic Resonance Center, UPMC, Kragujevac, PA USA; Internal Medicine, UPMC, Kragujevac, PA USA

## Background

Global longitudinal strain (GLS) and global circumferential strain (GCS) have emerged as important new markers of left ventricular (LV) function. Aortic stenosis (AS) creates pressure overload which produces LV hypertrophy and increased mass. Ejection fraction (EF) is the traditional parameter of LV systolic function, but the effects of increased LV mass on GLS and GCS are unclear. Our objectives were to: 1) assess the feasibility of a simple and novel feature-tracking speckle strain algorithm using standard CMR cine images in AS patients with CMR-derived LVEF as the reference standard for LV systolic function; 2) to assess whether left ventricular mass index (LVMI) alters the relationship between CMR-derived strain and LVEF.

## Methods

53 consecutive AS patients (85% with severe AS) who underwent CMR study (1.5T Siemens Magnetom Espree) and TTE within 15 days (median 6 days, IQR 0-15) were included in the analysis. AS severity was assessed by TTE as per societal guidelines. Feature tracking analysis of CMR cine images was performed using TomTec 2D Cardiac Performance Analysis software for measurement of GLS and GCS. Linear regression analysis was used for correlation of variables of interest.

## Results

The median age 69 years (IQR: 59-80 yrs), 59% were males. By TTE, the mean indexed aortic valve area was 0.47 ± 0.13 cm^2^/m^2^ and mean AV gradient of 34 ± 15 mmHg. By CMR, median LVEF=54% (IQR: 31-67%) and LV mass index=74 ± 19 g/m^2^. LVMI correlated weakly with GLS (r=0.35, p=0.01) but not with GCS (r=0.15, p=0.30). Correlation was high between LVEF and GLS (r=-0.74, p<0.0001, panel A) and GCS (r=-0.85, p<0.0001, panel B). To assess the impact of LVMI into the strain x LVEF relationship, patients were divided into 2 groups: low LVMI (mean: 62 ± 7 g/m^2^) or high LVMI (92 ± 16 g/m^2^). Regardless of the LVMI (low and high LVMI), both GLS (r=-0.79 and r=-0.74, p<0.0001) and GCS (r=-0.81 vs r=-0.85, p<0.0001) remained strongly correlated to LVEF.

## Conclusions

In patients with AS, novel feature speckle-tracking strain algorithm can easily measure important deformational changes from standard CMR cine images without need for dedicated acquisition and lengthy dataset post-processing. GLS and GCS correlated well with LVEF, regardless of the LVMI measured by CMR, which is precise and reliable. Understanding the determinants of these novel imaging parameters such as GLS and GCS will be necessary to establish their potential role into the risk-assessment and stratification of AS patients.

**Figure 1:**
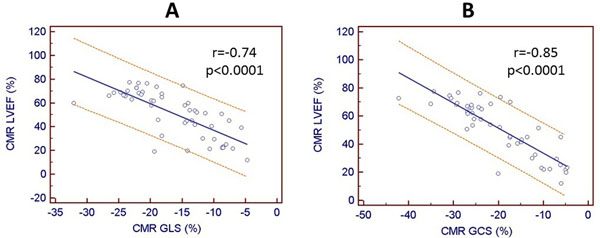
Spearman's correlations. 1a. Correlation between Echo and surgical findings. (correlation coefficient of 0.35). 1b. Correlation between CMR and surgical findings (correlation coefficient of 0.98).

